# Pancreatic Adenocarcinoma Treated With Irreversible Electroporation Case Report: First Experience and Outcome

**DOI:** 10.1097/MD.0000000000000946

**Published:** 2015-07-02

**Authors:** Francisco Javier Trueba-Arguiñarena, Diego Soto de Prado-Otero, Rodrigo Poves-Alvarez

**Affiliations:** From the Department of Radiodiagnosis (FJT-A); Department of Oncology (DSDP-O); and Department of Anesthesia and Postoperative Care, University Clinical Hospital, Valladolid, Spain (RP-A).

## Abstract

Irreversible electroporation (IRE) is a new nonthermal tumor ablation modality that induces apoptosis in the treated tissue without affecting collagen. Its use is particularly indicated for tumors involving major structures, such as encompassed or infiltrated vessels and/or ducts, which need to be preserved and hinder or preclude surgical resection. We report a 66-year-old male patient with locally advanced pancreatic adenocarcinoma, treated with IRE.

Two cycles of neoadjuvant chemotherapy with nab-paclitaxel and gemcitabine were administered. After these 2 cycles, IRE ablation was performed with a percutaneous transgastric access under general anesthesia. Later, 4 additional chemotherapy cycles were administrated.

At 48 hours of electroporation, blood tests were normal. On day 5, a computed tomography (CT) scan showed portal vein and celiac artery were normal in appearance. Three months later, a positron emission tomography (PET) scan showed disappearance of abnormal uptake in the pancreas and other sites. A 12-month follow-up the patient is disease free.

IRE opens a new way to treat tumors with involvement or proximity of neighboring structures. This procedure is more costly than other techniques and is not free of complications. The percutaneous transgastric access is feasible and without serious complications. In our case, complications were resolved and the patient presented a good short/medium-term outcome.

## INTRODUCTION

Irreversible electroporation (IRE) is a new, nonthermal tumor ablation modality that induces apoptosis in the treated tissue without affecting collagen.^1–8^ IRE is, therefore, particularly indicated for tumors involving major structures — such as encompassed or infiltrated vessels and/or ducts — that need to be preserved and hinder or preclude surgical resection. IRE is, of course, a local therapy, and therefore its indications do not include disseminated tumors. There is also a limit to the size of the tumors that can be treated with this type of ablation.

We report our first experience with percutaneous ablation of a locally advanced pancreatic adenocarcinoma by IRE and its medium-term outcome.

## CASE REPORT

**FIGURE 1:**
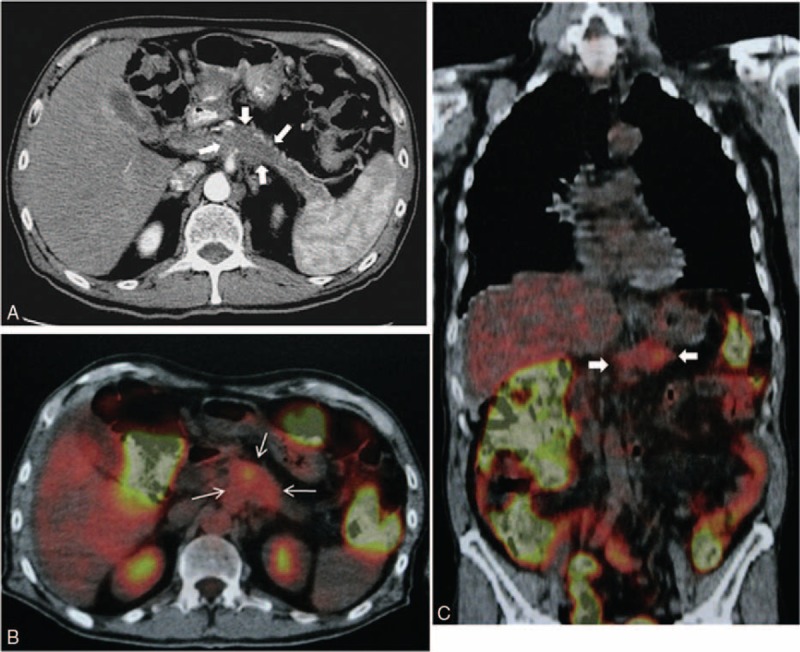
A, CT scan showing an ill-defined tumor in the pancreatic body (arrows). B and C, PET scan (axial and coronal images) showing abnormal uptake of F18-FDG in the site of the pancreatic tumor (arrows).

Our patient is a white 66-year-old male with wasting syndrome, normal blood tests, and normal tumor markers. A chest/abdomen computed tomography (CT) scan revealed an ill-defined, slightly hypodense mass in the pancreatic body, measuring 4 × 3 × 3 cm and encompassing the celiac artery (Figure 1A), as well as splenic and mesenteric vein thrombosis with abundant collateral circulation from intestinal venous return, and a patent portal vein.

A transgastric endoscopic ultrasound-guided biopsy confirmed the diagnosis of well-differentiated pancreatic adenocarcinoma.

A positron emission tomography (PET) scan (Figure 1B and C) showed diffuse abnormal uptake of F-18 fluoro-2-deoxyglucose (F^18^-FDG) in the pancreatic body.

Two cycles of neoadjuvant chemotherapy with nab-paclitaxel and gemcitabine were administered. Chemotherapy cycles consisted of nab-paclitaxel at a dose of 125 mg/m^2^ and gemcitabine 1000 mg/m^2^ on days 1, 8, and 15 every 28 days. Treatment tolerance was good; the main nonhematologic toxicity included grade 1 asthenia, grade 1 neuropathy, and grade 1 anemia; hematologic toxicity included grade 1 leukopenia. Cycles were given with no dose reduction or delay in chemotherapy. The reevaluation CT scan following neoadjuvant chemotherapy showed partial response of the pancreatic tumor, with no evidence of distant disease.

**FIGURE 2:**
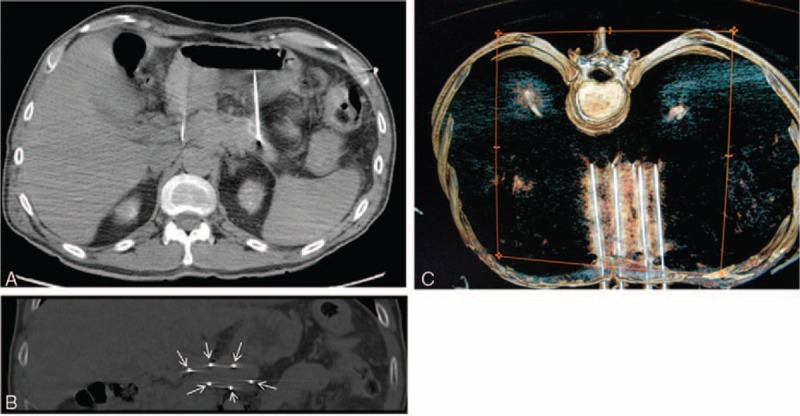
A, CT scan (axial view) showing 2 of the needles placed at the ends of the tumor. B, CT scan (coronal view) showing the 6 needles placed at the edge of the tumor (arrows). C, CT scan 3D (superior view) showing the 6 needles placed.

After these 2 cycles, and after the informed consent of the patient, IRE ablation was performed percutaneously under balanced general anesthesia with sevoflurane, fentanyl, and deep neuromuscular blockade. Six needles were placed surrounding the tumor through an anterior transgastric approach under CT guidance (Figure 2), and 90 high-voltage pulses were applied between the needles.

The patient had severe pain on waking and for 1 week, which during the first 3 days subsided with morphine, thereafter alternated with paracetamol. At 48 hours, blood tests showed completely normal amylase, lipase, tumor markers, white blood cells, etc.

On day 5, a follow-up CT scan was performed due to mild right abdominal discomfort; the only findings were free fluid in the right abdomen and edema in the wall of ascending colon, which was treated with diuretics. Splenic and mesenteric vein thrombosis persisted, but the portal vein was patent and the celiac artery was normal in appearance. No evidence of active bleeding or pneumoperitoneum was noted. No changes in the tumor image indicating the efficacy of the ablation were observed, except a slight increase in size of the treated area, probably by slight inflammation. Day 5 blood tests were normal.

On day 9, the patient experienced an episode of hematemesis with a few percent drop in his hematocrit, which was treated with a transfusion. No more bleeding episodes occurred.

Two weeks after ablation, the patient was discharged without analgesic medication.

Chemotherapy was subsequently continued with an additional 4 treatment cycles with the above-described regimen of nab-paclitaxel and gemcitabine, so that the patient received a total of 6 cycles. In the last 2 cycles, the dose of both drugs was reduced to 85% because of nonhematologic toxicity, mainly grade 2 asthenia.

**FIGURE 3:**
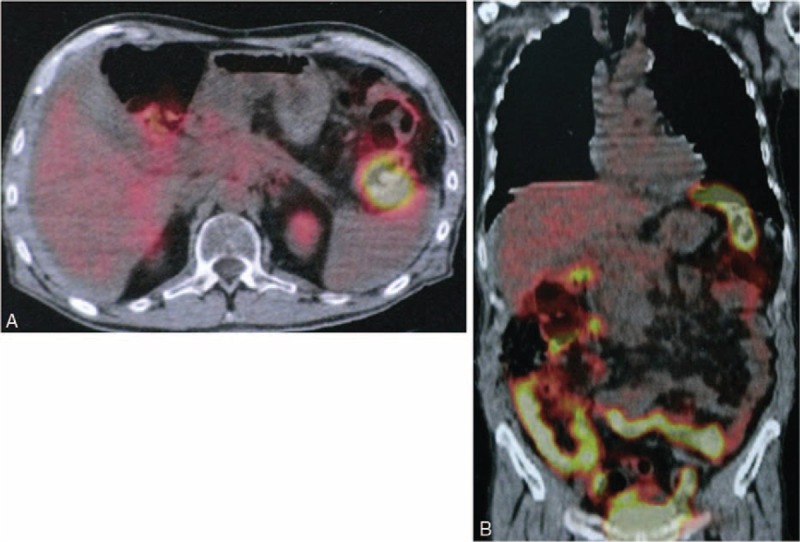
Follow-up PET scan (axial and coronal images) at 3 months after the procedure, showing disappearance of abnormal uptake in the pancreas.

A PET scan performed 3 months after the procedure displayed disappearance of abnormal uptake in the pancreas (Figure 3), and no other sites of uptake in the rest of the body. Currently, the follow-up period is 12 months and the patient is disease free, at home, has gained weight, and has no discomfort.

## DISCUSSION

IRE ablation opens a new field for the treatment of tumors that, because of their proximity to or involvement of neighboring structures, were excluded from treatment with current thermal ablation systems as they could also damage neighboring structures, which moreover hinder or preclude classic surgical approaches. Therefore, treatment was so far limited to chemotherapy and/or radiotherapy, generally with palliative results.

The treatment of pancreatic cancer is multidisciplinary. The fact that patient survival is poor due to distant relapse, even in localized tumors, is explained by dissemination in early stages. One strategy to improve survival is chemotherapy to prevent tumor spread or to treat small, still undetectable distant disease sites. Recently, Von Hoff and colleagues reported on a phase III clinical trial showing that nab-paclitaxel plus gemcitabine was superior to gemcitabine alone in metastatic disease.^9^ Other studies have subsequently reported the efficacy of this combination in the neoadjuvant setting, allowing tumor resection to be performed in a high percentage of patients.^10^ This is the reason why we chose this regimen for our patient. Although the duration of adjuvant therapy is not yet defined, we decided to administer 4 chemotherapy cycles after local treatment by electroporation. Thus, a total of 6 cycles were given, which is usually the recommended number of cycles in the adjuvant setting.^11^

Because IRE is a purely local treatment over the tumor, like other authors,^2,8^ in this case chemotherapy was administered pre- and post-IRE to try to control potential still undetectable scattered lesions.

In our patient, procedural complications included significant pain during the first days, similar to that of pancreatitis and requiring morphine for 1 week.

Another complication was the emergence of free fluid and edema in the wall of ascending colon, which we believe was due to a needle that damaged the collateral vein through which the venous drainage from this portion of the intestine passed until another collateral vein grew. (It should be remembered that the patient had preexisting mesenteric vein thrombosis, and his venous drainage passed through venous collaterals.) This complication was managed conservatively with diuretics.

A CT scan was performed a few days after the IRE but no shown alterations in the treated area which indicate whether IRE was effective, except for a slight enlargement that we believed as inflammatory type. However, this CT allowed excluding the existence of other vascular lesions in the celiac axis or portal vein by the punctures or the procedure. As the tumor was better detected in the PET scan before the ablation, it was decided to repeat a PET 3 months after the IRE to avoid false results as inflammatory changes and apoptosis in the treated area that can persist for up to 8 weeks postablation.^7^

Finally, another complication was the episode of hematemesis, which required only 1 transfusion and was related to the placement of 6 transgastric needles.

Ablation of pancreatic tumors with IRE has been performed commonly with open surgery, and with less frequency percutaneously, including a rear access to try to prevent punctures in other organs or structures.^12^ We believe a transgastric anterior access is feasible and without major complications due to the thick muscular wall of the stomach.

This ablation procedure is more costly than prior ablation techniques and is not without complications. In our patient, however, complications were not serious and were resolved with conservative measures. The short/medium-term outcome has been good, with apparent disappearance of the tumor, and opens a new door to the management of these malignancies.

Longer-term follow-up is of course necessary, the patient is disease free during 12 months of follow-up, and the initial results are very encouraging.
